# No impact of instructions and feedback on task integration in motor learning

**DOI:** 10.3758/s13421-020-01094-6

**Published:** 2020-10-08

**Authors:** Harald Ewolds, Laura Broeker, Rita F. de Oliveira, Markus Raab, Stefan Künzell

**Affiliations:** 1 Institute for Sports Science, Augsburg University, Universitätsstraße 3, 86159 Augsburg, Germany; 2 Institute of Psychology, German Sport University Cologne, Cologne, Germany; 3 School of Applied Sciences, London South Bank University, London, UK

**Keywords:** Task-integration, Multitasking, Implicit learning

## Abstract

This study examined the effect of instructions and feedback on the integration of two tasks. Task-integration of covarying tasks are thought to help dual-task performance. With complete task integration of covarying dual tasks, a dual task becomes more like a single task and dual-task costs should be reduced as it is no longer conceptualized as a dual task. In the current study we tried to manipulate the extent to which tasks are integrated. We covaried a tracking task with an auditory go/no-go task and tried to manipulate the extent of task-integration by using two different sets of instructions and feedback. A group receiving task-integration promoting instructions and feedback (N = 18) and a group receiving task-separation instructions and feedback (N = 20) trained on a continuous tracking task. The tracking task covaried with the auditory go/no-go reaction time task because high-pitch sounds always occurred 250 ms before turns, which has been demonstrated to foster task integration. The tracking task further contained a repeating segment to investigate implicit learning. Results showed that instructions, feedback, or participants’ conceptualization of performing a single task versus a dual task did not significantly affect task integration. However, the covariation manipulation improved performance in both the tracking and the go/no-go task, exceeding performance in non-covarying and single tasks. We concluded that task integration between covarying motor tasks is a robust phenomenon that is not influenced by instructions or feedback.

## Introduction

Task integration is a major factor in the study of dual-task performance, where studies claim that it can improve dual-task performance, depending on the task characteristics. When integrating tasks, people are thought to functionally combine the features from the main task and the secondary task, rather than processing the features of the two tasks separately. Some authors suggest that task integration is a natural principle of human processing since people have great difficulty processing two tasks separately, and so they would strive for task integration even if being instructed to perform two tasks (Röttger, Haider, Zhao, & Gaschler, 2017; Schmidtke & Heuer, 1997). The task-integration hypothesis proposes that when two tasks contain a sequence, participants do not process these sequences separately, but rather perceive the two sequences, and thus the tasks, as one combined sequence comprised of sequence elements of both tasks (Schmidtke & Heuer, 1997).

Task integration can, however, be either detrimental or beneficial to performance. A combined sequence is usually more complex than the sequences within either task, and as such task integration can cause costs and prevent participants from learning the single sequences within each task (Schmidtke & Heuer, 1997). On the other hand, task integration has been shown to be beneficial, especially for motor learning. One circumstance that has been shown to foster integration and optimal performance is covariation of task features in the serial reaction time task (SRTT; see Nissen & Bullemer, 1987) and an auditory task, demonstrated by Schmidtke and Heuer (1997). In their study participants pressed buttons in response to visual and auditory stimuli. When stimuli of the visual and auditory task alternate and both tasks contain an equal number of elements in a predictable sequence, the combined sequence is a lot less complex than when the number of elements is unequal. This type of covariation of tasks (combining visual and auditory stimuli) led to better dual-task performance than when tasks did not covary (Schmidtke & Heuer, 1997). Covariation in this case provided predictability to the task, where each element of the combined sequence can be predicted by the previous element, even though it belonged to the other task. In contrast to tasks without covariation, task integration of covaried tasks should enhance dual-task performance with covaried tasks.

While most studies on task integration employ some kind of covariation between tasks to study task integration (de Oliveira, Raab, Hegele, & Schorer, 2017; Röttger et al., 2017; Schmidtke & Heuer, 1997), some authors view task integration differently. Ruthruff, van Selst, Johnston, and Remington (2006) tried to demonstrate task-integration by comparing single-task and dual-task training effects of non-covarying tasks. They examined dual-task performance with an auditory-vocal and visual-manual task after training only task 1, only task 2, or dual-task training. The authors argued that evidence for task-integration would be obtained if dual-task practice led to greater improvements than single-task practice. Conversely, if single-task practice was more effective, task automatization would be the cause for dual-task cost reduction. Ruthruff and colleagues found no indication of task-integration. The mechanism behind the beneficial effect of task-integration in this study would not have been covariation, as described in the studies before. Instead the authors argued that dual-task training caused better task scheduling, leading to less interference caused by a response-selection bottleneck (Pashler, 1994). Task integration of non-covarying tasks may also take place, and indeed play an important role in the performance of many everyday motor tasks. In the present study, however, since a beneficial effect of task-integration has thus far mostly been demonstrated in covarying tasks, we chose to use this method to test whether task-integration can be manipulated via instructions.

Whether people really do integrate tasks into a single task, and to what extent, may partly depend on how the tasks are presented (besides covariation). Both instructions and type of feedback (Halvorson, Wagschal, & Hazeltine, 2013; Srna, Schrift, & Zauberman, 2018) may contribute to task integration and better performance, eventually even leading to the conceptualization of two tasks as a single task. Conceptualization, the perception of dual tasking and its impact on subsequent performance, has been investigated by Srna et al. (2018). In their study participants were asked to solve two puzzles that through instructions and context were either presented as being a single task (task-integrating instructions) or two different tasks (task-separating instructions). The task features did not covary. Participants receiving the task-separating instructions rated the tasks more strongly as multitasking, and performed better, than participants in the task-integrating group. However, given that the task features did not covary, the attempt to integrate tasks was harder than keeping them separate, and so task-integrating instructions might have had a negative effect in this study. Nevertheless, since their instructions were effective in changing the way participants perceived the tasks, we adapted their instruction to fit with the covariation manipulation we used in the current study. We also used a similar questionnaire to that in Srna et al. (2018) to obtain a measure of how participants conceptualized the tasks.

In a study closely related to ours, de Oliveira, Raab, Hegele, and Schorer (2017) investigated whether task integration also benefits motor learning, combining a tone-counting task with a repeated segment in a manual tracking task. They covaried the two tasks by placing tones shortly before occurrences of turns in the tracking path so that tones made turns predictive. Note that with this manipulation, the tone task predicted the tracking task but not vice versa, thus tasks did not become interdependent as in the SRTT of Schmidtke and Heuer (1997), where tones and visual stimuli were both predictive of each other. De Oliveira et al. (2017) found no benefits of covariations for implicit learning when comparing the “integration group” to a group performing only single tasks or non-covaried tasks. However, other research examining implicit learning and task integration in continuous tasks is sparse. In the present study we adapted the paradigm by de Oliveira et al. (2017), but we exchanged the counting task for a motor-response task and we used a within-group design to test whether implicit learning under task-integration conditions would transfer to single and random dual-task conditions. In addition, we focused on the question of whether the extent of task integration could be manipulated by instructing the tasks differently, as in Srna et al. (2018). In one group, task-separating instructions emphasized the existence of two tasks and explained that the two tasks were distinct, with the idea that this would give a stronger perception of performing two tasks at the same time and reduce between-task interactions (Fischer & Plessow, 2015). The task-integration group performed the same tasks, but instructions framed them as a single task. The instructions (see *Methods*) were formulated to be as contrasting as possible on the dual-task versus single-task dimension, i.e., a strong focus on the existence of two tasks for the task-separation group while all wording referring to the existence of two tasks was avoided for the task-integration group. In addition to the two instructions, we manipulated feedback. While the task-integration group received two scores, one for each task, the task-separation group received a single score for both tasks together. To also contrast the environment as much as possible, without altering the actual task demands for both groups, we included different familiarization phases. While the task-integration group practiced the tasks together from the very beginning, the task-separation group had to practice the tasks separately.

In sum, we hypothesized that covarying two tasks would foster task integration, which would improve dual-task performance reflected by superior tracking performance and lower reaction times. In addition, we hypothesized that this effect would be stronger for the group receiving task-integrating instructions and one feedback score compared to the group receiving task-separating instructions and two separate feedback scores. Furthermore, we expected implicit learning to be preserved. The results of our study would add to the understanding of how people conceptualize tasks with the same task features that only differ in instructions and feedback.

## Methods

### Participants

Forty students participated in the experiment for course credit (22 female; *M*_*age*_ = 20.3 years, *SD* = 2.2). Sample size was based on de Oliveira et al. (2017), who found an effect size of 0.24 with a sample size of 30 in a between-subjects design. Participants had normal or corrected-to-normal vision and no experience in tracking. Participants were assigned to a task-integration group (n = 20) or a task-separation group (n = 20). Two participants of the task- integration group were later removed from data analysis because of problems with data recording.^1^

Before the start of the experiment, participants gave informed consent. Ethics approval was obtained from the local ethics committee and the study conformed to the principles of the Declaration of Helsinki.

### Apparatus and material

The experimental setup was adapted from de Oliveira et al. (2017), in which implicit learning was demonstrated. Participants were seated in front of a 24-in. computer screen (144 Hz, 1,920 × 1,080 resolution). The cursor in the tracking task was controlled by a T.16000M FCS joystick. Positional data were recorded at 120 Hz on a Windows 7 Computer. Stimuli of the auditory task were presented through Sony stereo headphones and participants responded by pressing the pedal on an f-pro USB-foot switch (9 cm × 5 cm).

### Task and display

**Fig. 1:**
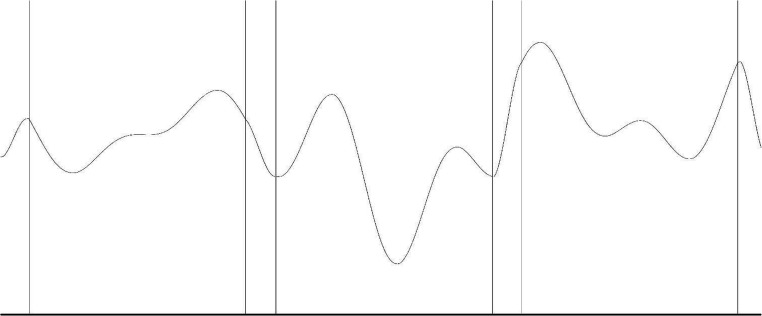
An example of a fictional path used in the tracking task for illustration purposes. The target square moved from left to right along the path depicted. Target tones were placed immediately before reversal points, signaling an upcoming turn; in addition, distractor tones were placed randomly. During the experiment participants could not see the path or the vertical lines depicted. The small areas between the three sections indicate zones that were used to connect the three segments; data were not collected there

The tracking task entailed pursuing a red target square of 19 × 22 pixels with a joystick-controlled white cross of equal dimensions from the left side of the screen to the right side of the screen. Only vertical movement of the cursor was user-controlled, horizontal tracking of the target occurred automatically. The path of the target followed a wave created from three segments of equal length where no segment could repeat within a single trial. The formula to create the segments was taken from Wulf and Schmidt (1997):

$$ f(x)={b}_0+\sum \limits_{i=1}^4{a}_i\sin \left(i\bullet x\right)+{b}_i\cos \left(i\bullet x\right) $$

with *a*_*i*_ and *b*_*i*_ being a randomly generated number ranging from -4 to 4 and *x* in the range of [0, 2π] (see Fig. 1). For this experiment, 32 segments similar in length and number of edges were selected. Twenty segments were selected to give each participant, which was repeated every trial during the training phase. As in Künzell, Sießmeir, and Ewolds (2016), each participant per group received their own repeating segment, ensuring that practice effects were not due to difficulty differences between segments or between segments of the two groups (Chambaron, Ginhac, Ferrel-Chapus, & Perruchet, 2006; Wulf & Schmidt, 1997). The two random segments on each trial were chosen so that each occurred an equal number of times. Three groups were created that differed in positioning of the repeated segment. The repeating segment was positioned at the start, in the middle, or at the end of the training trials, since segment positioning might be a possible confounder of tracking performance. Trial duration was between 24 and 37 s.

The auditory task was a go/no-go reaction time task and required pressing a pedal upon hearing high-pitched tones (1,086 Hz) while ignoring low-pitched tones (217 Hz), as adopted from Schmidtke and Heuer (1997). In total, there were ten target and ten distractor tones per trial, and target tones were positioned 250 ms before a reversal point in the curves in the training phase.

### Procedure

Participants read the instructions on the screen before the experiment. Instructions for the task-separation group emphasized the existence of two tasks to induce the perception of performing a dual task: “Task 1: You sit in front of a computer screen. The start screen will display a red square on the left side of the screen. You will also see a white cursor that you can control with the joystick. To align the cursor with the target square, move the joystick forwards. When they are aligned you can press the button on the front of the joystick and the experiment will begin. After the start the red square will move up and down. It is your task to use the cursor to follow the target square to the right side of the screen. Track the target square as accurately as possible. To do this you only move the joystick vertically. The horizontal movement is automatic. After every trial the starting screen appears again. By clicking the joystick, you can start the next trial. After every five trials you will see the amount of points you scored in the tracking task and in the tone task. The higher the score the better your performance was. The points will be used by the researcher.”

In contrast, task-integrating instructions presented the tasks as being one, they explained the connection between the tones and tracking task, and avoided all mention of multitasking to induce the perception of performing a single task. Instructions for the task-integration group were: “You will perform one task while sitting in front of a computer screen with a pedal below the desk. The start screen will display a red square on the left side of the screen. You will also see a white cursor that you can control with the joystick. To align the cursor with the target square, move the joystick forwards. When they are aligned you can press the button on the front of the joystick and the experiment will begin. After the start the red square will move up and down. It is your task to use the cursor to follow the target square to the right side of the screen. Track the target square as accurately as possible. To do this you only move the joystick vertically. The horizontal movement is automatic. At the same time place your preferred foot on the pedal below the table. You will hear two different tones through the headphones. The higher tone will indicate there will be reversal of direction of the target square on the screen; confirm with a pedal press as quickly as possible that you hear the higher tone, ignore the lower tones. Every five trials you will see a score that reflects how accurately you have done this task. The higher the score the better the performance. The points will be used by the researcher.”

During the familiarization phase, the task-separation group first practiced the tracking task and auditory task separately, then as a dual task. Practice for the task-integration group only involved dual-task trials. Throughout the experiment participants received feedback after every five trials. For the task-integration group this feedback was a single score on the screen constructed from scores of both tasks. Performance on each task was converted so that lower root mean square error (RMSE) and lower reaction times both result in a higher score; in addition the reaction time score was multiplied by a factor so that it contributed about equally to the integrated score as the RMSE score. The task-separation group received a score for each task separately but calculated the same way. To start each trial, participants moved the cursor to the target square and pressed a button on the joystick. Instructions were repeated every two blocks to reinforce the manipulation.

The experiment took place over 3 days. On the first day participants were familiarized with the tasks and did two training blocks. For both groups training blocks consisted of 20 trials with the repeating segment and with the tones predicting reversals in the tracking path. On the second day, 1 week later, they did two further training blocks and the test block. In the test block, all participants performed five single-task (ST) trials in each task and then five dual-task (DT) trials as in the training blocks. At the end, participants performed five dual-task trials without the covariation between the tracking task and tone task. Instead, tones were presented randomly. On the third day, the test block was repeated as a retention test.

After the retention test explicit knowledge of the repeating segment was checked for with the same questionnaire used in Ewolds et al. (2017). The questions were: (1) Did you notice anything special during the experiment? (2) Was there something that helped or hindered you while performing the tracking? (3) Did you apply any rules? (4) Did you notice anything special concerning the path of the target? (5) The target followed a certain path. Did you notice any segments in this path? (6) There were three segments in the path, the first, the middle, and at the last segment. One of these segments was always repeated. Did you notice? (7) Which segment was the repeated segment, the first, the middle, or the last segment?

Additionally, for the task-separation group, we tried to find out whether they noticed the connection between the tracking task and the tone task by asking the following additional questions: (8) Did you notice anything about the combination of the tracking task and tone task? (9) Did you discover a pattern between the tracking and tone task? (10) If so, what was the pattern? Finally, as in Srna, Schrift, and Zauberman, (2018), we asked participants of both groups to rate on a scale of 1 to 7 how strongly they felt that they had been doing a single task or two tasks, with a 1 indicating strong feelings of single tasking and a 7 indicating strong feelings of dual tasking.

### Data analyses

Tracking performance was measured by the RMSE, calculated from the difference in position between the target square and the user-controlled cursor. Performance on the repeated segments was compared to average performance of the two random segments. Reaction times and errors were recorded for the tone task.

To test for learning effects during the *training phases*, RMSE, reaction times, and errors were submitted to a 4 × 2 × 3 × 2 mixed analysis of variance with within-subjects factor Training Block (four training blocks), Segment (Repeating vs Random), segment Position (placement of the repeating segment), since this might influence learning (Zhu et al., 2014), and between-subjects factor Group (Task integration vs. Task separation).

To test for effects on RMSEs in the *test block and retention* test we performed a 3 × 2 × 2 × 2 mixed analysis of variance with within-subjects factors Condition (Single task, DT with covariation, DT without covariation), Segment (Repeating vs. Random), Test (Test block vs. Retention block) and between-subjects factor Group (Task integration vs. Task separation) and Position (placement of the repeating segment). For the reaction times we did a 3 × 2 × 2 mixed analysis of variance with within-subjects factor Condition (Single task, DT with covariation, DT without covariation), Test (Test block vs. Retention block) and between-subjects factors Group (Task integration vs. Task separation). Finally, to test whether reaction times were quicker during repeated tracking segments we performed a 2 × 2 ANOVA with Segment (Repeating vs. Random) and Condition (DT covariation vs. DT without covariation). A Greenhouse-Geisser correction was used when the assumption of sphericity was violated. To test differences in perceptions of dual tasking versus single tasking for the two groups a Mann-Whitney test was performed.

## Results

We expected participants to improve both RMSEs and reaction times as a result of covarying the tasks. Although these effects were not clear during the training phase, they were apparent in the Test Block and Retention Test. We could not find evidence that giving task-integrating or task-separating instructions influenced the degree to which tasks were integrated subjectively, nor did it affect dual-task performance (see below).

### Training blocks

**Fig. 2:**
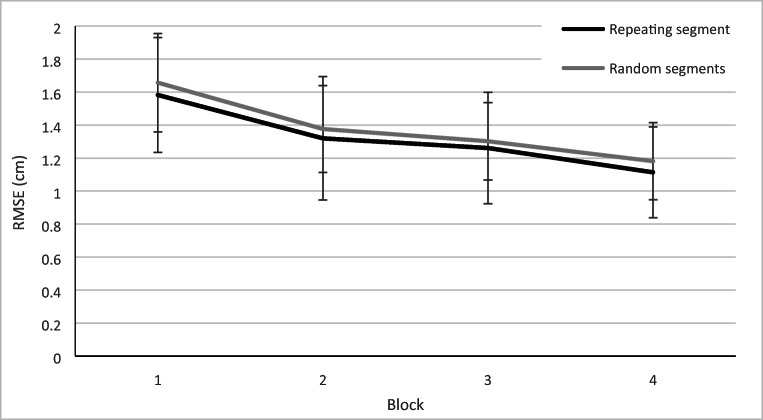
Root mean square error (RMSE) improved from the first to the last training block**.** Errors bars represent the standard deviation of the mean

**Fig. 3:**
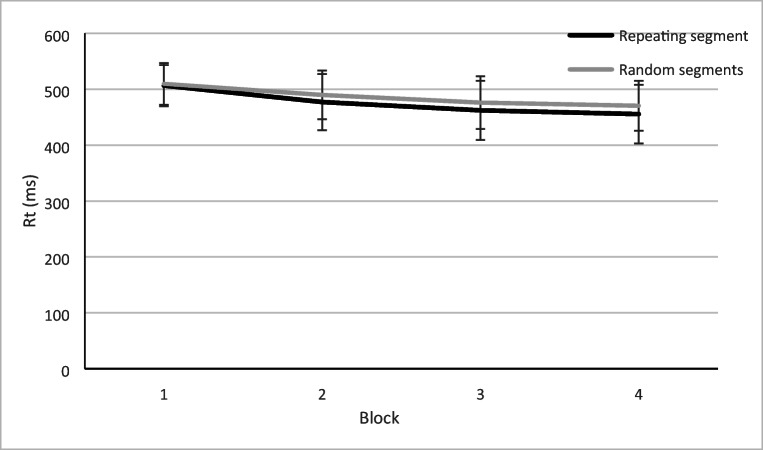
Reaction times (RTs) during the training blocks. Errors bars represent the standard deviation of the mean

Improvements in tracking, *F*(2.28, 72.95) = 97.99, *p* < .001, η_p_^2^ = .754, and reaction times, *F*(3, 96) = 42.81, *p* < .001, η_p_^2^ = .572, were observed during the training blocks (see Fig. 2 and Fig. 3). Errors also reduced with training, *F*(1.72, 54.96) = 8.22, *p* = .001, η_p_^2^ = .204, although they were already rare with just 0.27 errors per trial in Block 1. No implicit learning of the constant segment could be demonstrated by using RMSEs as we did not find a Block × Segment interaction, *F*(3, 96) = .463, *p* = .709, η_p_^2^ = .014. The position of the repeating segment did not have an effect on RMSE, *F*(2, 32) = 0.33, *p* = .721, η_p_^2^ = .045. Reaction times did improve more during repeating segments than on random segments, *F*(3,96) = 4.80, *p* = .004, η^2^ = .130.

### Test block and retention test

**Fig. 4:**
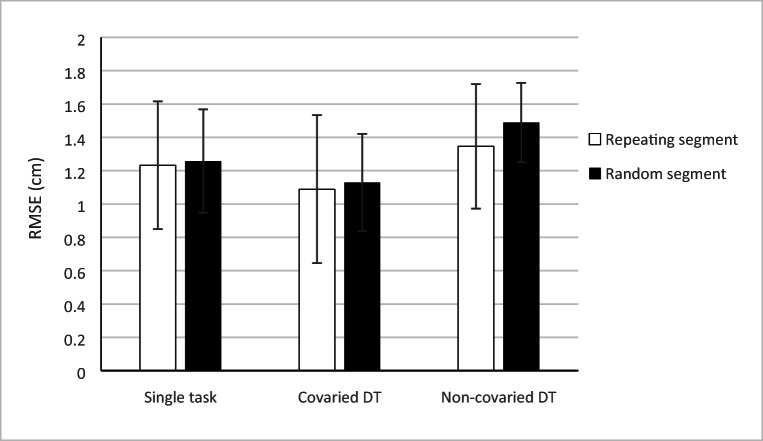
Tracking performance (RMSE) of the test block comparing single task, covaried dual task (DT) and non-covaried DT. Regardless of segment, performance on the covaried DT surpassed performance on the non-covaried and single-task trials. Performance on the repeated segment was significantly better in the non-covaried DT condition only. Error bars represent the standard deviation of the mean

The results of the ANOVA on RMSEs revealed a significant effect of Condition, *F*(2, 64) = 120.71, *p* < .001, η_p_^2^ = .790, with the best performance in the DT covariation condition, then the single-task condition, and worst performance on the DT without covariation condition, all *p* < .001 (see Fig. 4).

Tracking performance on repeating segments was better than on random segments, as indicated by a main effect of Segment, *F*(1, 32) = 9.45, *p* = .004, η_p_^2^ = .228. A significant Segment × Condition interaction, *F*(1.60, 51.06) = 20.81, *p* < .001, η_p_^2^ = .394, indicated that the difference in performance between the repeating and random segments was mainly found in the DT without covariation condition. Neither was a difference between the test block and retention test found, *F*(1, 32) = 1.71, *p* = .201, η_p_^2^ = .051, nor a significant effect of Group, *F*(1, 32) = .36, *p* = .553, η_p_^2^ = .11 or Segment-Position, *F*(2, 32) = .33, *p* = .721, η_p_^2^ = .020.

**Fig. 5:**
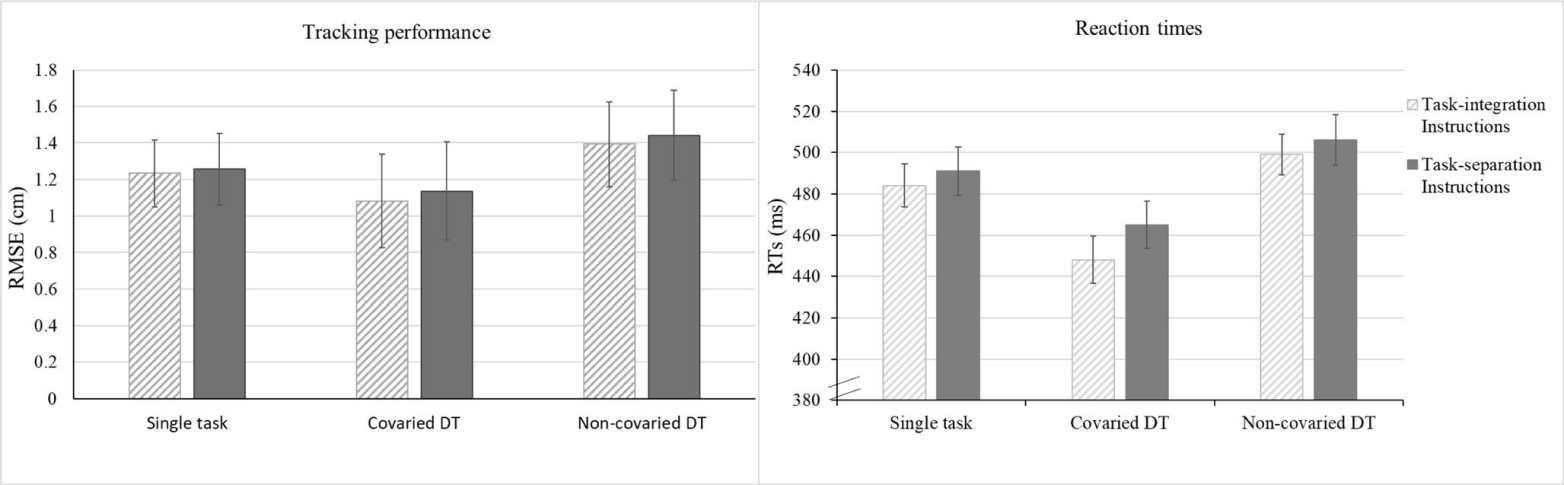
Tracking (RMSE) and reaction-time (RT) performance during the test block. Best performance was found in the covaried DT condition. No effect of instruction was found. Error bars represent the standard deviation of the mean

For reaction times we found a significant effect of Condition, *F*(2, 64) = 41.31, *p* < .001, η_p_^2^ = .564. Post hoc comparisons using the Bonferroni procedure revealed that the best reaction time performance was found in the covaried DT, then the single-task condition, and worst performance in the non-covaried DT condition, all *p* < .001 (see Fig. 5).

A significant effect of Test, *F*(1, 32) = 10.74, *p* = .003, η_p_^2^ = .251 indicated that reaction times improved from the test block (*M =* 481 ms, *SE = 59 ms*) to the retention test (*M* = 471 ms, *SE = 55 ms*). We found no effect of Group, *F*(1, 32) = .30, *p* = .588, η_p_^2^ = .009, so the task-integration and task-separation groups did not differ significantly. A significant effect of Position, *F*(2, 32) = 4.72, *p* = .016, η_p_^2^ = .228, indicated that position of the repeating segment matters. Post hoc paired comparisons with Bonferroni correction revealed that when the repeating segment was at the end, reaction times were 46 ms faster than when it was in the middle, *p* = .014. Testing reaction time during repeating and random tracking we could not find a main effect of Segment, *F*(1, 32) = 1.73, *p* = .197, η_p_^2^ = .051, but there was a significant Segment × Condition effect, *F*(1, 32) = 13.20, *p* = .001, η_p_^2^ = .292, which indicated that in the covariation dual task, reactions were 16 ms faster during repeated tracking compared to random tracking, while in the non-covariation condition no difference between repeated and random tracking paths could be found.

### Interviews

**Fig. 6:**
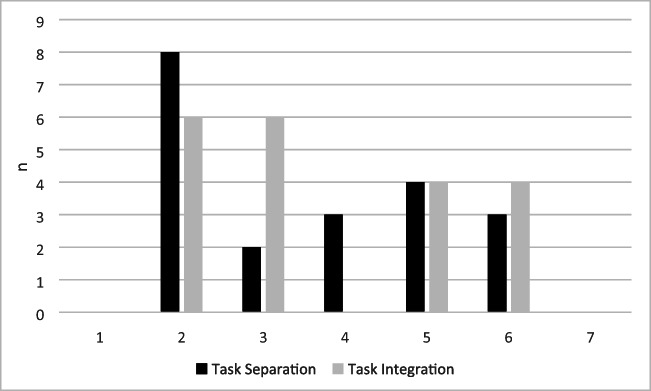
Frequency distributions per group (task integration vs. task separation) of the ratings participants gave to the question “How strongly do you feel that you were doing one task or two tasks?” With '1' indicating very strong feelings of single tasking and '7' very strong feelings of dual tasking

Analyses of the interviews showed that eight participants had explicit knowledge of the repeating segment. Overall, when forced to make a choice, 63% of the participants correctly named the position of the repeating segment. Of the 20 participants in the task-separation group, 14 nevertheless noticed this covariation. Perception of dual tasking versus single tasking did not differ between the groups, as frequency distributions to question 11, *“How strongly do you feel that you were doing one task or two tasks?”* did not significantly differ between the task-integration (*mean rating = 3.7*) and the task-separation group (*mean rating = 3.6*), *U* = 188, *p* = .758 (see Fig. 6).

## Discussion

The goal of the current study was to investigate whether task integration likely resulting from covariation of the tasks improved multitasking, and whether it enabled implicit learning in dual tasking. Our results showed that covariation improves performance on both the tracking and the reaction-time task. Performance in covaried dual tasks was better than in non-covaried or single tasks. Since the tones announced the changes in the tracking curve, lower tracking errors might not be surprising; however, reaction times were also faster in the covaried dual-task condition, even though the tracking path did not predict the occurrence of sounds. Yet one might argue that whenever the target square approaches the edges of the screen the occurrence of a turning point and thus a target tone becomes more likely, so the boundaries of the monitor entailed some predictability in this regard. Better performance in tracking and reaction times indicate that participants likely integrated the two tasks, which is why we adhere to the position that task integration can be beneficial to dual-task performance and not necessarily costly. We were unable to demonstrate that this effect was stronger for the group receiving task-integrating instructions and feedback, so we suggest that the task features were the main driver for task integration, rather than instructions or feedback.

Reaction times were more sensitive than RMSEs to the predictability in the tracking task during training (see Figs. 1 and 2), demonstrating that the benefits of predictability within one task can transfer over to the other task when the tasks can be integrated. The current experiment confirms the conclusion by Röttger et al. (2017) that implicit sequence learning during dual tasking can be preserved when tasks covary, showing this is also true for more complex motor tasks. The extent to which sequence knowledge was used seemed to differ for the different conditions, with the largest advantage of implicit knowledge shown in the most difficult condition, the dual task without covariation, indicating that implicit information can be used flexibly, and is not necessarily tied to the conditions under which it was acquired. However, the opposite was found for reaction times, where participants benefited from the repeating segment only in the covarying dual task, which was the easiest condition. Possibly this discrepancy is caused by a shift in priority to the tracking task in the most difficult condition (Broeker et al., 2018).

As in de Oliveira et al. (2017) we found better performance in the task-integration condition than in the single-task condition. Better dual-task performance than single-task performance is otherwise rare, but has been found in highly automatized behaviors such as saccades during pointing movements (Huestegge & Koch, 2014) and expert performance in sports (Beilock, Carr, MacMahon, & Starkes, 2002). It is unlikely that the effects in our study are due to task automatization, since in that case performance on the non-covaried dual task would not differ much from the covaried dual task, which it did. Instead when one task informs the other tasks, it becomes more predictable allowing for advanced motor planning.

Our study does not provide an explanation about how dual-task costs are reduced, but it contributes to the literature by showing that covariation is beneficial to first and secondary task performance. Whether a covariation manipulation removes dual-task effects or alters the tasks in different ways is difficult to determine, but it does at least show that the benefit from the predictability provided by the secondary task offsets any dual-task performance deficits caused by having to respond to a secondary task. One finding that speaks for the absence of dual-task performance deficits, and the realization of a fully integrated task, is that performance on the covaried task surpassed that of single-task tracking. The structural similarity might have caused the two tasks to be processed as a single unit. Since dual-task costs are often explained in terms of tasks competing for limited resources or a bottleneck in processing, reducing a dual task to an integrated single task would remove the theoretical limitation to dual-task performance. So far, however, the dual-task literature indicates that task integration is only helpful when structural similarities exist. Learning is impaired when tasks do not covary, likely because there is no relation between successive elements (Schmidtke & Heuer, 1997). In the case of two unrelated tasks, the preservation of task boundaries is helpful, as shown by the effectiveness of instructions emphasizing *task separation* in preserving implicit learning (Halvorson et al., 2013; Röttger et al., 2017).

We tested task integration by instructions and type of feedback, and whether this would improve performance measures. The degree of task integration was not influenced by instructions or by feedback scores, in line with the finding that participants’ perception of performing single versus dual tasks did not significantly differ between groups. This was surprising since the instructions were formulated to contrast each other as much as possible, and because similar instructions have been found to be effective before (Halvorson et al., 2013; Srna et al., 2018). Since the majority of the participants in the task-separation group discovered the covariation between the tasks, it is likely that task perception was influenced by the task features. Simply the fact that two different effectors and stimuli streams are used might make some people classify this situation as a dual task, while others may be convinced through the covariation that it is more like a single task. It is difficult to theoretically establish what constitutes a single task or a dual task (Freedberg, Wagschal, & Hazeltine, 2014; Künzell et al., 2018; Rogers & Monsell, 1995), it may be even more difficult to induce a subjective experience of single tasking or dual tasking with covarying tasks.

### Limits of our study and future research

The degree to which tasks are actually integrated is difficult to measure, performance measures alone are helpful but likely insufficient since dual-task training without covariation will also reduce dual-task costs. Interviews as in our study provide a performance-independent measure, but suffer from subjectivity and they do not consider that the way participants perceive tasks during performance might not be explicitly clear to participants themselves at the interview. A promising avenue for research would be to focus on the neural mechanisms behind dual tasking. Studies have shown that different brain areas are active during dual tasking and single tasking (Garner & Dux, 2015; Watanabe & Funahashi, 2014). Garner and Dux (2015) found that training of non-covarying dual tasks promotes a separated perception of the tasks in the brain, accompanied with performance improvements. Hypothetically long-term training of covarying tasks could lead to more integrated perceptions of tasks. This might answer the question of whether task integration causes the formation of a single task (Künzell et al., 2018) or whether it allows for hyper-efficient task scheduling (Salvucci & Taatgen, 2008).

While it seems tempting to assume that task integration is only possible or beneficial when certain features of tasks are covaried, it is not straightforward to determine where this covariation must come from. In the current study the covariation was quite obvious for most participants. Motor learning often involves integrating several sub-components to work as a whole, but how exactly these components are integrated is not always obvious. Finding out how tasks may be combined to integrate them effectively and beneficially is a promising area of research (Franz, Swinnen, Zelaznik, & Walter, 2001; Klapp & Jagacinski, 2011; Swinnen & Wenderoth, 2004) and one with immense practical applications in our current world.

### Conclusion

Data from the current study show that the perception of dual tasking versus single tasking with covarying tasks may be difficult to influence, but this perception may not matter for task performance. Nevertheless, because the type of covariation used was quite arbitrary, and will probably not be encountered with many real-world tasks, there is a possibility that manipulation of perceptions of less subtle covarying tasks may improve task performance. In a world where multitasking is unavoidable, providing a covariation between tasks and presenting them in a way in which that covariation can be fully exploited could significantly increase productivity.

## Footnotes

1 The problems with data recording concerned the auditory task. These two subjects showed no reaction-time recordings for large sections of the experiment. This is likely caused by participants not lifting their foot up enough after pressing the pedal, which prevents subsequent reactions from being registered. This problem was spotted during the practice trials and corrected, but it re-surfaced later in the experiment. We are sure that this problem did not exist for the remaining participants because very few reaction-time errors were made.
